# Mitigation of Structural Vibrations in Sensitive Audio Devices: A Study on Isolation Materials for Lightweight Turntables

**DOI:** 10.3390/ma18112617

**Published:** 2025-06-03

**Authors:** Aleksandra Sawczuk, Bartlomiej Chojnacki

**Affiliations:** Department of Mechanics and Vibroacoustics, AGH University of Krakow, Mickiewicza Av. 30, 30-059 Cracow, Poland; asawczuk@student.agh.edu.pl

**Keywords:** turntable vibration isolation, low-frequency resonance control, precision audio systems, lightweight turntable engineering, vibration damping materials

## Abstract

Effective vibration isolation is critical for minimizing the transmission of unwanted mechanical energy from a source to its surrounding environment, especially in precision systems, where even minor disturbances can degrade performance. This study addresses the challenge of low-frequency vibration transmission in lightweight, high-sensitivity audio devices such as turntables with masses below 10 kg. Traditional vibration mitigation strategies—primarily based on increasing system mass to raise the resonant frequency—are unsuitable for such systems due to weight constraints and potential impacts on operational dynamics. Previous studies have identified a critical resonance range of 5–15 Hz, corresponding to the tonearm and cartridge assembly, where transmitted vibrations can compromise signal fidelity and cause mechanical degradation. This research aims to develop an effective and universal vibration isolation solution tailored for lightweight turntables, focusing on external isolation from structural vibration sources such as furniture and flooring. To achieve this, a two-stage experimental methodology was employed. In the first stage, the excitation method with the use of a hammer tapping machine was evaluated for its ability to simulate real-world vibrational disturbances. The most representative excitation methods were then used in the second stage, where the isolation performance of various materials and systems was systematically assessed. Tested isolation strategies included steel springs, elastomeric dampers, and commercial linear vibration isolators. The effectiveness of each isolation material was quantified through spectral analysis and transfer function modeling of vibration acceleration data. The results provide comparative insights into material performance and offer design guidance for the development of compact, high-efficiency anti-vibration platforms for audio turntables and similar precision devices.

## 1. Introduction

With the proliferation of digital music distribution platforms such as Spotify and Apple Music, the use of physical media, including CDs and vinyl records, has markedly declined over the past two decades [1,2]. Nevertheless, recent market analyses reveal a significant resurgence in the popularity of vinyl records and analog turntables [3,4]. This renewed interest is evidenced by a consistent increase in sales figures and a growing number of album releases in vinyl format. In response to the expanding mainstream consumer market within the general electronics sector, manufacturers have introduced turntables that are both cost-effective and accessible to a broader audience.

Beyond addressing a consumer-level problem, this study is rooted in a broader scientific objective: to develop and experimentally validate a generalizable framework for low-frequency vibration isolation in lightweight, compliant mechanical systems. The turntable serves here not merely as a commercial device, but as a representative platform for investigating dynamic interactions between structural components and environmental disturbances. The main scientific contributions of this research are threefold:

- The development of a scalable experimental method for low-frequency vibration analysis based on multi-point acceleration measurement and chirp z-transform (CZT) spectral analysis,
- The empirical validation of this method using different classes of vibration isolators with known dynamic parameters,
- The integration of simplified mechanical modeling to interpret observed system behavior, particularly the discrepancy between theoretical and real-world isolation thresholds.

These contributions support broader engineering domains where precise, cost-effective vibration control is required, ranging from laboratory instrumentation to consumer-grade precision devices.

However, given the inherently delicate and precise nature of turntables as audio reproduction equipment, efforts to reduce production costs have frequently resulted in design simplifications and the use of lower-quality materials. A critical consequence of these compromises is an increased sensitivity to both internal and external sources of vibration. Previous studies have demonstrated that turntables are particularly vulnerable to low-frequency vibrations, notably within the 3–6 Hz range, which is associated with plate resonances, and the 8–15 Hz range, corresponding to tonearm–cartridge resonances [5,6,7].

Vibrations affecting turntable performance can be attributed to two principal sources: internal and external. Internal vibrations primarily originate from the motor operation and the mechanical rotation of the plate. External vibrations arise from environmental and human-induced activities such as footfall, furniture movement, or nearby vehicular traffic, with these disturbances often transmitted through building structures to the turntable [8,9,10]. Such vibrations, detectable at the chassis level, have been shown to audibly degrade playback quality, with previous research confirming that the resulting distortions are perceptible even to untrained listeners.

To address these challenges, high-end turntable manufacturers have implemented a variety of engineering solutions. These include enhancements to cartridge compliance, the adoption of advanced tonearm designs (e.g., curved tonearms), the utilization of high-stiffness modern materials, and the incorporation of sophisticated vibration isolation systems to attenuate both external excitations and internally generated disturbances [11,12]. While effective, these measures substantially increase manufacturing costs and are typically confined to premium turntable models priced above EUR 5000. Consequently, there is a growing demand for alternative, low-cost vibration isolation solutions suitable for more affordable turntable models [13,14,15].

The objective of the present study is to evaluate the feasibility of applying effective vibration isolation techniques to lightweight, low-cost turntables. Achieving low-frequency vibration isolation in such models presents unique challenges, particularly due to their reduced mass—typically less than 10 kg—whereas effective isolation ideally begins at frequencies as low as 5 Hz. In contrast, high-end turntables, which often weigh between 50 and 60 kg, inherently facilitate improved isolation performance through greater mass and mechanical damping.

This research addresses two primary goals:

- The development of a repeatable and efficient methodology for measuring vibrations affecting lightweight turntables.
- The investigation of low-frequency vibration isolation solutions suitable for application to systems with reduced mass.

The structure of the remainder of this paper is as follows: Section 2 describes the measurement techniques and isolation materials evaluated; Section 3 presents the results of the vibration acceleration measurements, including frequency spectrum analysis and transfer function characterization; Section 4 provides a detailed discussion of the findings; and Section 5 offers conclusions along with suggestions for future research directions.

## 2. Materials and Methods

Prior to selecting appropriate vibration isolation solutions, it was essential to establish accurate and repeatable methods for evaluating their effectiveness. This section describes the proposed methodology for conducting precise acceleration measurements at critical points on the turntable. Section 2.2 presents the selection criteria and characteristics of the vibration isolation materials investigated, while Section 2.3 outlines the analytical techniques employed to assess their isolation performance.

### 2.1. Lightweight Turntables Measurement Methods

The measurements were conducted in a room with a concrete floor, where the turntable was placed directly on the surface. The experimental setup consisted of the following equipment:

- RME Fireface UFX sound card (Haimhausen, Germany),
- Norsonic Nor277 hammer tapping machine (Tranby, Norway),
- B&K type 2694 preamplifier (Naerum, Denmark),
- Acceleration sensors (PCB 352C22, PCB 288D01, PCB 356B18—New York, NY, USA),
- Computer for data acquisition and processing.

Prior to the experiments, all vibration sensors were calibrated using a Svantek SV110 (Warsaw, Poland) calibrator (reference acceleration: 10 m/s^2^). The lightweight accelerometers (PCB 352C22), each with a mass not exceeding 10% of the corresponding measured component, were mounted at key locations: on the cartridge, tonearm, and turntable plate [16,17]. An impedance sensor (PCB 288D01) was installed on the substrate, while a triaxial sensor (PCB 356B18) was mounted on the chassis, with the Z-axis aligned vertically.

**Figure 1:**
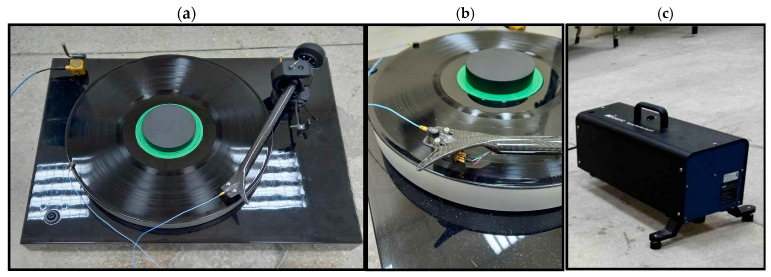
Photographs of the measurement setup: (**a**) general view of the turntable; (**b**) placement of the acceleration sensor on the cartridge; (**c**) positioning of the tapping machine for vibration excitation.

Vibration excitation was introduced using a Nor277 hammer tapping machine, simulating external disturbances such as footfall- or furniture-induced vibrations transmitted through the floor. Each excitation event lasted 15 s. The placement of the accelerometers, as well as the excitation method, are illustrated in Figure 1.

Initial measurements were performed without any supplementary vibration isolation to assess the baseline performance of the turntable’s original feet. Subsequently, the original feet were removed, and various isolation materials (described in Section 2.3) were positioned beneath the turntable at four locations: two at the front corners and two at the rear.

When analyzing vibrations induced by the tapping machine, a primary challenge was the identification of individual impact events within the recorded time signal. To address this, a windowing technique was applied, enabling the isolation of discrete impulses from the broader excitation signal. Five successive impulses were manually selected (corresponding to the number of hammers in the tapping machine), and their frequency spectra were subsequently averaged [16]. This approach ensured the repeatability and consistency of the analyzed data.

**Figure 2:**
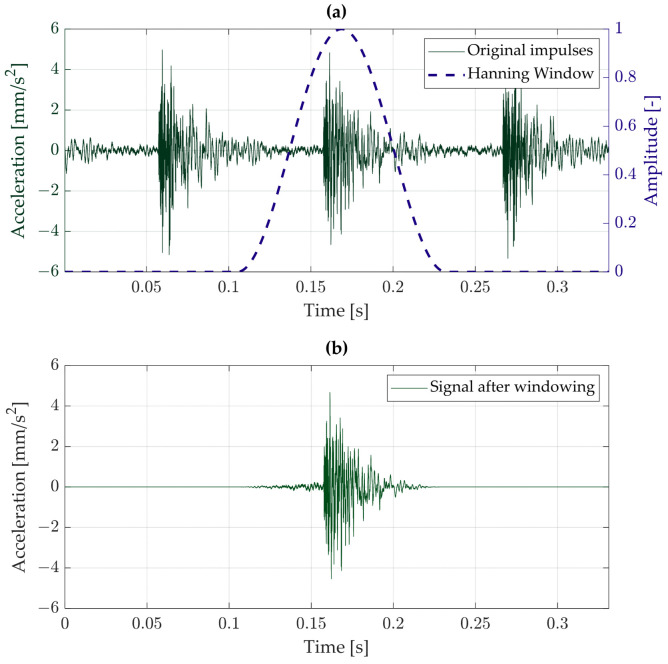
Example vibration signal registered on the cartridge: (**a**) full signal with several registered impulses, (**b**) single impulse after time window application.

A Hanning window was applied to each selected impulse segment, as illustrated in Figure 2. Figure 2a shows the raw impulse signals together with the applied Hanning window profile, while Figure 2b presents the isolated impulse after windowing. For consistency, the beginning of each windowed segment was aligned to the same relative position within each impulse. The window length was set to 6100 samples. Additionally, a band-pass filter with a passband of 2–100 Hz was applied to the data to focus the analysis on the relevant vibration frequency range [18,19,20].

It is noted that the application of windowing inherently reduces spectral resolution due to the limited number of samples per segment. To mitigate this effect, the chirp z-transform (CZT) was employed. The CZT allows spectral analysis within a specified frequency range at an arbitrary resolution, providing enhanced accuracy, particularly in the critical low-frequency bands [21,22].

It is noted that during all measurement procedures, the turntable was powered off and not playing music. This was performed to isolate the influence of externally transmitted structural vibrations and exclude internal vibration sources from the analysis.

### 2.2. Analysis Methods

To evaluate and compare the performance of the selected materials for low-frequency vibration isolation, two primary analysis methods were employed. The first method involved calculating the averaged frequency spectrum in selected bands, a widely accepted standard for assessing vibration levels and the effectiveness of vibroisolation systems. The second method was based on transfer function analysis, applied in two distinct configurations. The transfer functions used in this study were derived from the Fourier spectrum values calculated for each impulse separately. After applying the time window, the results were averaged across all impulses recorded in the time signal to smooth the final spectrum results. In the first configuration, the vibration spectrum of the turntable supported by an isolation material was compared to that of the unisolated reference setup. This relationship is defined by Equation (1):

(1) $$H_{1}(f)=10\log\left(\frac{s_{v}(f)}{s_{r}(f)}\right)$$

where

*s_v_*—acceleration signal Fourier spectrum measured on the turntable element with the vibroisolation mounted,

*s_r_*—acceleration signal Fourier spectrum measured on the turntable element without the vibroisolation mounted turntable placed on solid blocks.

The second analyzed transfer function spectrum was used to quantify the amount of vibration energy transmitted from the ground to the device and is defined as follows (2):

(2) $$H_{2}(f)=10\log\left(\frac{s_{t}(f)}{s_{g}(f)}\right)$$

*s_t_*—acceleration signal Fourier spectrum measured on the turntable element,

*s_g_*—acceleration signal Fourier spectrum measured on the ground.

Each of the acceleration signal spectrums was treated as an average spectrum, which was calculated separately from all the impulses recorded in the measured signal. This approach allowed for smoother measurement data and improved repeatability. Ultimately, over 2000 spectrums were averaged to perform the transfer function calculations.

### 2.3. Vibroisolation Materials

The selection of suitable vibration isolation materials for this application presented a significant challenge, primarily due to the requirement for effective isolation at very low frequencies. Achieving low-frequency vibroisolation necessitates the use of materials or systems capable of accommodating large static displacements. When combined with the relatively low mass of the isolated object, this requirement demands a highly compliant suspension system.

**Figure 3:**
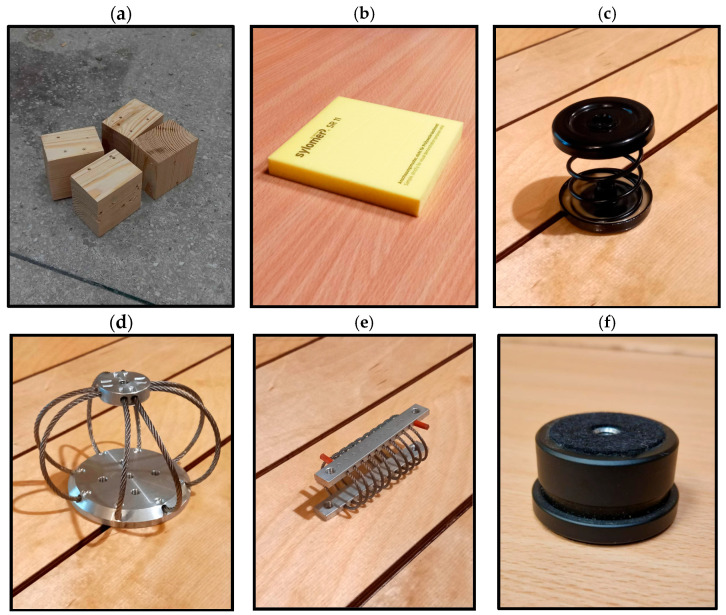
Vibroisolating devices evaluated for low-frequency isolation of a lightweight turntable: (**a**) rigid solid wood blocks used as the reference configuration; (**b**) SR11 Sylomer elastomer (Getzner); (**c**) Isotop MSN KTL 1 spring isolator; (**d**) AMC WireRope Type B isolator; (**e**) PGFUN TR0-25 line-rope isolator; (**f**) standard factory-installed turntable feet.

The design parameters for the isolation system were established as follows: a load mass of 10 kg, four isolators to ensure adequate stability, and a target system resonance frequency of approximately 5 Hz. Given the limited knowledge regarding the exact vibration characteristics affecting turntables in practical use, a broad range of isolation strategies was considered. These included spring-based systems, elastomeric materials, and wire rope isolators. The selected vibroisolation configurations are presented in Figure 3.

The key parameters utilized in the design of the vibroisolation systems are summarized in Table 1. To achieve the target resonant frequency, the elastomer-based solution was constructed using four stacked layers of SR11 material, each with a thickness of 12.5 mm. The resonant frequencies for all measured systems were calculated based on the datasheets and nomographs provided by the manufacturers of each vibration isolation material, considering the desired vibroisolation frequency range based on expected static deflection [18,23]. Variations observed in the calculated resonant frequencies among the tested configurations are attributed to differences in the dynamic stiffness properties of the respective materials.

## 3. Results

To evaluate the effectiveness of the vibration isolation systems, measurement data from sensors mounted on the cartridge and the plate of the turntable were selected, as these components are the most critical for minimizing the transmission of unwanted vibrations.

### 3.1. Spectral Analysis—Cartridge

**Figure 4:**
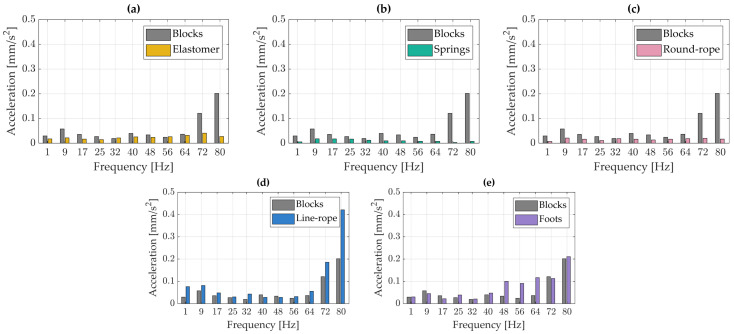
Spectral analysis of the vibrations registered on the cartridge: (**a**) blocks vs. elastomer, (**b**) blocks vs. springs, (**c**) blocks vs. round-rope isolator, (**d**) blocks vs. line rope-isolator, (**e**) blocks vs. foots.

Figure 4 presents the spectral analysis of vibration accelerations measured on the cartridge. Each peak in the plot corresponds to vibration acceleration within frequency bands of approximately 8 Hz, reflecting the original resolution of the Fourier transform applied to the windowed signal. All results obtained using various vibration isolation solutions were compared against the reference measurements taken with the turntable mounted on rigid wooden blocks. A noticeable increase in vibration acceleration amplitudes is observed at above approximately 64 Hz.

In the low-frequency range, a local maximum is observed at around 9 Hz. This peak is likely associated with a resonance in the cartridge–tonearm system, resulting from the interaction between the effective mass of the tonearm and the compliance of the cartridge.

Across the entire analyzed frequency range, almost all vibration isolators contributed to a reduction in vibration acceleration amplitudes. In the 32–64 Hz bands, the greatest attenuation was achieved using the spring-based isolators. The elastomer proved to be the most effective within the 17–32 Hz range; however, around 56 Hz, the vibration amplitudes were slightly higher than those recorded with the turntable mounted on rigid wooden blocks, indicating a localized increase in vibration levels. The round-rope isolator exhibited the most favorable performance in the 25–32 Hz bands.

Figure 4 further shows that the use of a line-rope isolator resulted in an increase in vibration acceleration amplitudes across nearly the entire frequency range. Only a slight reduction in vibrations was observed with the turntable’s original feet, suggesting that while they provide structural support, they lack effective vibration isolation capabilities.

### 3.2. Spectral Analysis—Plate

**Figure 5:**
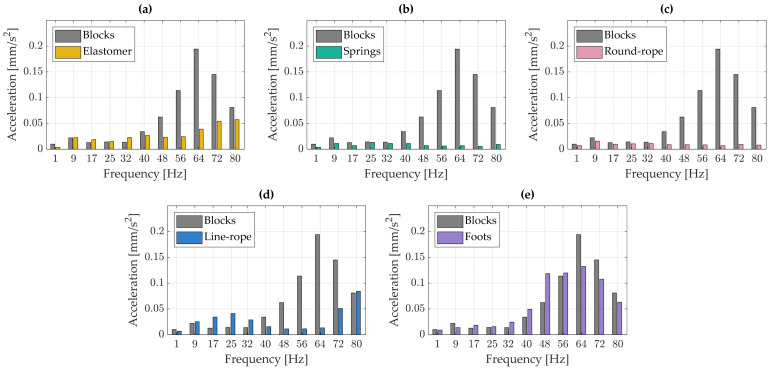
Spectral analysis of the vibrations registered on the plate: (**a**) blocks vs. elastomer, (**b**) blocks vs. springs, (**c**) blocks vs. round-rope isolator, (**d**) blocks vs. line rope-isolator, (**e**) blocks vs. foots.

For the turntable plate, spectral vibration analysis was conducted using the same methodology as applied to the cartridge. The results obtained under impact excitation are presented in Figure 5. In this case, the line-rope vibration isolator exhibited significantly better damping performance, particularly within the 40–72 Hz frequency range, achieving greater vibration reduction than the elastomer. These findings contrast with the results observed for the cartridge, where the line-rope isolator was among the least effective solutions. Consistent with previous observations, the spring-based isolators provided the most effective vibration attenuation overall; however, in this instance, the round-rope isolator demonstrated a performance level comparable to that of the springs.

### 3.3. Transfer Function Analysis—Vibroisolation Vs. Stiff Placement—Cartridge

**Figure 6:**
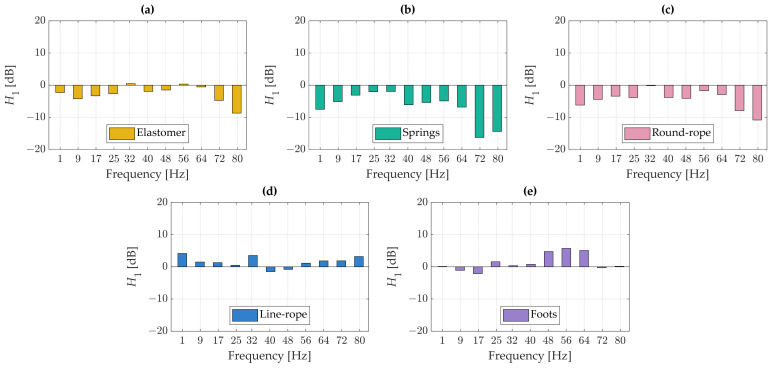
Transfer function analysis on the cartridge—vibroisolation vs. stiff placement: (**a**) elastomer, (**b**) springs, (**c**) round-rope isolator, (**d**) line rope-isolator, (**e**) foots.

Figure 6 presents the transfer function *H*_1_. According to the adopted interpretation method, transfer function values below zero indicate effective vibration attenuation, whereas positive values signify an amplification of vibrations.

Analysis of the transfer function reveals that the line-rope isolator demonstrates the poorest vibration damping performance, as it contributes to increased vibrations across nearly the entire frequency range, showing attenuation (negative values) in only two bands. The turntable’s original feet performed worst within the 48–64 Hz range, and across other bands, they either failed to reduce vibrations or exhibited only minimal damping effectiveness. The best results were achieved with the spring-based isolators, which provided the highest level of vibration reduction across both low- and high-frequency ranges. The transfer functions corresponding to the elastomer and the round-rope isolator were similar in both shape and magnitude; however, the elastomer demonstrated lower damping effectiveness, particularly within the lowest frequency bands.

### 3.4. Transfer Function Analysis—Ground Vs. Cartridge

**Figure 7:**
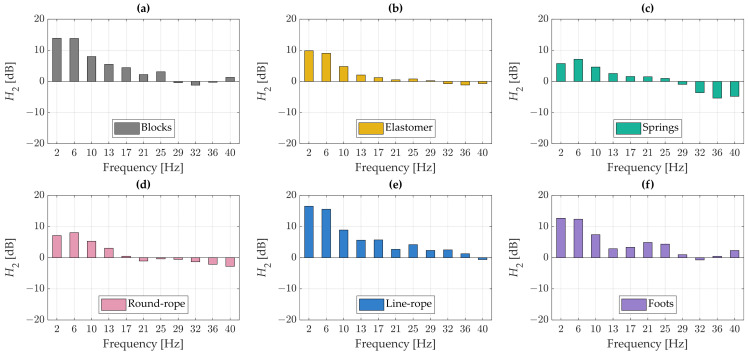
Transfer function analysis—ground vs. cartridge: (**a**) blocks, (**b**) elastomer, (**c**) springs, (**d**) round-rope isolator, (**e**) line rope-isolator, (**f**) foots.

Figure 7 presents the transfer function *H*_2_, calculated as the ratio of the vibration spectra measured by the sensors located on the cartridge and on the ground. Negative values of the transfer function indicate that the vibration levels at the protected component (the cartridge) are lower than those at the ground.

At the lowest frequencies, the vibration amplitudes of the cartridge are observed to exceed those of the ground. This effect is expected, as the cartridge is a lightweight and highly compliant element, making complete suppression of its vibrations inherently challenging. Nevertheless, for nearly all types of vibration isolators tested, the transfer function values in this frequency range are lower compared to the reference case where the turntable was placed directly on rigid wooden blocks.

## 4. Discussion

The experimental results clearly demonstrate that most vibration isolators contributed effectively to the reduction in vibration amplitudes across a wide frequency spectrum. However, the damping performance of each material exhibited significant dependence on the frequency range and the specific turntable component under observation.

Spring-based isolators consistently achieved superior vibration attenuation, providing effective damping across both low and high-frequency bands. This finding indicates that springs offer a highly versatile and robust solution for vibration isolation in lightweight turntables, owing to their favorable dynamic compliance and ability to accommodate low resonant frequencies without excessive stiffness.

In contrast, elastomer-based isolators exhibited more selective performance. While elastomers were effective in specific frequency bands—particularly within the mid-frequency range—they showed limitations outside of these bands, with occasional amplification effects observed, notably around 56 Hz. This behavior suggests that elastomeric materials may be more suitable for applications targeting a narrower, well-defined frequency band rather than broadband isolation. Their viscoelastic nature, while beneficial in certain damping regimes, introduces stiffness variations that can negatively affect performance at both very low and higher frequencies.

The line-rope isolator was observed to induce an increase in vibration amplitudes across most of the measured frequency range, particularly when applied to lightweight components such as the cartridge. This adverse effect may be attributed to improper deformation behavior under load, a phenomenon likely related to the isolator’s structural properties and nonlinear dynamic response. The mismatch between the isolator’s stiffness characteristics and the mass of the protected element likely resulted in dynamic amplification rather than attenuation, highlighting the critical importance of matching isolation system properties to the mechanical characteristics of the supported structure.

The factory-installed feet of the turntable, primarily designed to provide basic mechanical support, were found to offer minimal vibration attenuation. Although they contribute to the overall structural stability of the device, they lack the damping and compliance necessary to mitigate vibrational disturbances effectively. This result underscores the need for aftermarket or integrated vibration isolation solutions in mass-market turntables where cost-driven designs often neglect precise vibration control.

Furthermore, the comparative analysis between different components—specifically the cartridge and the turntable plate—revealed that the effectiveness of a given isolation material is not universal across all subsystems. Isolators that performed favorably for the cartridge, such as elastomers, did not necessarily deliver the same degree of vibration reduction for the plate, and vice versa. These discrepancies emphasize that the dynamic response of each component, influenced by factors such as mass, compliance, and natural frequency, plays a decisive role in the overall isolation effectiveness. Hence, achieving optimal performance requires a system-specific design approach rather than relying on a single isolation solution for the entire structure.

To enhance the technical validation of our findings, we conducted a comparative analysis of the measured isolation threshold frequencies. These frequencies are defined as the points at which the magnitudes of the transfer functions begin to drop below the levels recorded for the “blocks” case, which serves as the reference for the non-vibroisolated turntable.

Although the nominal resonance frequencies for the isolators ranged from 3.8 Hz to 5.2 Hz (calculated based on material stiffness and mass), with the expected vibroisolation range starting at 8 Hz, the experimental transfer function results (Figure 6 and Figure 7) consistently demonstrated that effective vibration attenuation did not occur until approximately 10 Hz. Below this frequency, most isolators exhibited positive or near-zero differences in transfer functions when compared to the “blocks” case, indicating either vibration amplification or minimal damping.

This discrepancy can be attributed to several practical factors:

1. The lightweight and compliant structure of the turntable, which causes dynamic amplification near the resonance of the isolators.
2. The non-ideal damping characteristics of the materials, particularly under real-world loading conditions.
3. The multi-degree-of-freedom behavior of the system, where the effective response is influenced by the interaction between the tonearm, cartridge, and plate masses—not just a simple single-degree-of-freedom model.

However, once the excitation frequency exceeded approximately 10 Hz, all tested isolation systems began to consistently reduce transmitted vibration energy, as evidenced by the decrease in transfer function magnitudes. This observed threshold aligns with prior research on vibration isolation in low-mass systems, which suggests that practical attenuation tends to begin well above the theoretical natural frequency due to complex system dynamics.

The findings of this study contribute to a broader scientific inquiry into vibration control in low-mass, compliant systems. The observed threshold for effective isolation—consistently around 13 Hz across all isolator types—deviated from theoretical resonance values derived from static stiffness and mass. This discrepancy highlights the limitations of simplified lumped-parameter models in accurately predicting isolation onset in systems with multiple interacting degrees of freedom and nonlinear damping behavior.

In summary, while all tested isolation strategies improved performance relative to the baseline case of rigid mounting, the spring-based systems emerged as the most effective and broadly applicable solution for lightweight turntables. The study highlights the necessity of careful material selection and system tuning, particularly for applications where mass and dynamic behavior vary significantly among different structural elements.

An important limitation of this study is that internal vibration sources, such as motor rotation or stylus-tracking forces during playback, were not included in the analysis. These sources may alter the dynamic response of the system, and future work will include measurements under operational conditions to assess isolator effectiveness in real-use scenarios.

## 5. Conclusions

This research comprehensively addressed the challenge of achieving low-frequency vibration isolation in lightweight, cost-effective turntables. Utilizing a structured two-stage experimental design—including realistic excitation methods, precision acceleration measurements, and advanced spectral analysis techniques such as windowing and chirp z-transform (CZT)—this study systematically evaluated the performance of various isolation strategies suited for turntables with masses below 10 kg.

The main findings of the investigation are summarized as follows:

- Springs (ISOTOP MSN 1 KTL) provided the most effective and consistent vibration attenuation across all analyzed frequencies, especially excelling in the critical 8–15 Hz and 32–64 Hz bands.
- The Sylomer SR11 elastomer showed good isolation performance in mid-frequency ranges (17–32 Hz) but revealed limitations at low and high frequencies, with occasional amplification effects around 56 Hz.
- Round-rope isolators (AMC WireRope Type B) demonstrated moderate damping performance, being particularly effective on the heavier turntable plate but less so on the lighter cartridge.
- Line-rope isolators (PGFUN TR0-25) generally increased vibration amplitudes across most frequency bands, suggesting that they are poorly suited without further adaptation.
- Factory-installed turntable feet exhibited minimal vibration isolation, providing structural support but failing to meaningfully attenuate external vibrations.

Beyond these specific results, a broader analysis reveals important trends and considerations. Vibration isolation effectiveness was found to be highly dependent not only on the material properties of the isolators but also on the characteristics of the turntable subsystems. The cartridge and tonearm system, owing to their low mass and high compliance, proved particularly sensitive, often amplifying low-frequency vibrations despite the use of isolators. Meanwhile, the heavier turntable plate responded more predictably, with clear benefits observed when using compliant materials such as springs and rope isolators.

Transfer function analysis confirmed that at very low frequencies (below 10 Hz), all tested configurations exhibited some degree of amplification, a phenomenon inherent to lightweight precision structures where dynamic interaction with external excitations cannot be fully suppressed. Nonetheless, when compared to the baseline scenario of rigid mounting, most isolation strategies achieved notable reductions in vibrational energy transmitted to the sensitive components of the system.

Importantly, the study demonstrated that spring-based isolation systems offer a broadly applicable and highly effective solution for enhancing vibration performance in lightweight turntables without imposing excessive cost or complexity. Elastomeric materials may provide targeted benefits for mid-frequency disturbances but require careful system-level tuning to avoid adverse side effects. Wire-rope isolators, while effective in some contexts, displayed inconsistencies that make them less attractive for highly sensitive audio applications without further optimization.

The experimental procedures developed in this research—including the detailed multi-point acceleration measurement setup and dual transfer function framework—provide a robust and replicable methodology for future evaluations of vibration isolation systems in lightweight, high-precision devices. The research demonstrates that, despite low system mass, effective vibration mitigation is achievable using properly selected materials—especially spring-based isolators—if paired with a precise analytical and experimental approach. Importantly, the methodology combines controlled excitation, high-resolution spectral analysis, and dual transfer function modeling to identify and interpret the dynamic behavior of compliant systems. While developed using a turntable context, the approach is broadly applicable to fields such as precision instrumentation, acoustic isolation in lab settings, and biomedical device stabilization. Future work will extend this experimental framework to include dynamic simulations and hybrid damping systems, enabling co-design of isolation and structural elements in next-generation lightweight devices. In this way, the research opens a practical and theoretical path toward robust vibration isolation strategies across engineering domains. In conclusion, this study establishes that with appropriate selection and tuning of isolation materials, effective low-frequency vibration mitigation can be achieved, even in low-mass, cost-sensitive audio turntables. This opens promising avenues for the broader democratization of high-fidelity analog playback equipment, allowing more consumers to experience the benefits of superior vibration control. Future work will focus on dynamic modeling, the exploration of hybrid isolator designs combining springs and viscoelastic materials, and the long-term stability testing of selected systems under operational stresses to ensure durability and sustained acoustic performance.

## Figures and Tables

**Table 1:** Material properties of used vibroisolators.

Variant	Used Code Name	Datasheet Name	Maximum Static Load	Dynamic Stiffness	Depth (Unloaded)	Calculated Resonant Frequency
(a)	Blocks	Raw material blocks	-	-	50 mm	-
(b)	Foots	Built-in turntable foots	-	-	30 mm	-
(c)	Springs	ISOTOP MSN 1 KTL	3.6 kg	1.8 N/mm	60 mm	3.8 Hz
(d)	Round-rope	AMC WireRope Type B	2.0 kg	9.1 N/mm	60 mm	4.3 Hz
(e)	Line-rope	PGFUN TR0-25	2.5 kg	1.9 N/mm	40 mm	5.1 Hz
(f)	Elastomer	Sylomer SR11	3.8 kg	20.1 N/mm	50 mm	5.2 Hz

## Data Availability

The original contributions presented in this study are included in the article. Further inquiries can be directed to the corresponding author.

## References

[1] Serazio M. (2021). What We Can Learn from the Revival of the Vinyl Record. Bus. Horiz..

[2] Barnett K. (2020). The Vinyl Revival: A Cultural and Economic Reassessment. J. Pop. Music. Stud..

[3] The Recording Industry Association of America (RIAA) (2023). America, R.I.A.A. of Year-End 2022 Music Industry Revenue Report.

[4] Osborne R. (2012). Vinyl: A History of the Analogue Record.

[5] Kilmanas R., Rabinow J. (1982). Tonearm Geometry and Frequency-Modulation Distortion-and Discussion. J. Audio Eng. Soc..

[6] Rother P. (1977). The Aspects of Low-Inertia Tone-Arm Design. J. Audio Eng. Soc..

[7] Anderson C.R. (1979). A Vibration-Stabilizer System for Phonograph Reproduction. J. Audio Eng. Soc..

[8] Nakai G.T. (1973). Dynamic Damping of Stylus Compliance/Tone-Arm Resonance. J. Audio Eng. Soc..

[9] Ladegaard P. (1977). Audible Effects of Mechanical Resonances in Turntables.

[10] van den Dobbelsteen M. (2008). The Tables Are Turned—A Study of Developments in Mainstream Audio Replay and the Emergence of a Vinyl Subculture. Master’s Thesis.

[11] Jones M., Lewis R. (2019). Modern Tonearm Designs and Materials for Improved Audio Playback. Appl. Acoust..

[12] Lavigne T., Stewart G. (2017). The Effects of Low-Frequency Vibrations on Turntable Audio Performance. J. Audio Eng. Soc..

[13] Miles J. (2016). The Beginner’s Guide to Vinyl: How to Build, Maintain, and Experience a Music Collection in Analog.

[14] Qu Z., Yao Y. Analysis and Measurement of Wobble Error on Simulation Turntable. Proceedings of the 1st International Symposium on Systems and Control in Aerospace and Astronautics 2006.

[15] Li Y., Wang J., Zhang X. (2022). The Research about Application of Quasi-Zero Stiffness Vibration Isolator in Launch Turntable. J. Sound Vib..

[16] Idczak J., Chojnacka K., Rubacha J., Kamisiński T. (2024). Investigation of Impact Sound Transmission through Various Mechanical Connectors of Lightweight Structures. Vib. Phys. Syst..

[17] Chojnacki B., Chojak A., Binek W., Idczak J., Pawlik J. (2024). Numerical Modeling of the Elementary Metamaterial Cells for the Sound-Absorbing Structure Preparation. Vib. Phys. Syst..

[18] Cieplok G., Wójcik K. (2020). Self-Synchronization of Drive Vibrators of an Antiresonance Vibratory Conveyor. J. Theor. Appl. Mech..

[19] Pawlik P. (2019). Single-Number Statistical Parameters in the Assessment of the Technical Condition of Machines Operating Under Variable Load. Eksploat. Niezawodn. Maint. Reliability.

[20] Korbiel T., Rubacha J. (2020). Analysis of the Vibration Measurement and Reduction Possibilities in Linear Infrastructure for Vacuum Rail Technology. New Trends Prod. Eng..

[21] Wszołek T., Mleczko D., Pawlik P., Kłaczyński M., Małecki P., Stępień B. (2022). Experimental Verification of the Usefulness of Selected Infrasound and Low-Frequency Noise Indicators in Assessing the Noise Annoyance of Wind Turbines. Vib. Phys. Syst..

[22] Litak G., Iwaniec M., Iwaniec J. (2017). Milling Gate Vibrations Analysis via Hilbert-Huang Transform. Proc. ITM Web Conf..

[23] Michalczyk J., Cieplok G. (1999). Wysokoefektywne Układy Wibroizolacji i Redukcji Drgań.

